# Physical and Physiological Demands of Official Beach Soccer Match-Play in Relation to Environmental Temperature

**DOI:** 10.3390/sports13040118

**Published:** 2025-04-14

**Authors:** Thiago Carvalho, Vincenzo Rago, João Brito, Priscyla Praxedes, Marco Abreu, Davi Silva, Sara Pereira, Magni Mohr, Ivan Baptista, José Afonso

**Affiliations:** 1 Centre of Research, Education, Innovation, and Intervention in Sport (CIFI2D), Faculty of Sport, University of Porto (FADEUP), 4200-450 Porto, Portugal; priscylapraxedes@hotmail.com (P.P.); profmarcoabreu@hotmail.com (M.A.); davizirt@hotmail.com (D.S.); sarasp@fade.up.pt (S.P.); jneves@fade.up.pt (J.A.); 2 National Youth Sports Institute, Singapore 397778, Singapore; vincenzo.rago@live.com; 3 Portugal Football School, Portuguese Football Federation, 1495-433 Oeiras, Portugal; joao.brito@fpf.pt; 4 CIPER, Faculty of Human Kinetics, University of Lisbon, 1649-004 Lisbon, Portugal; 5 Department of Sports Science and Clinical Biomechanics, SDU Sport and Health Sciences Cluster (SHSC), Faculty of Health Sciences, University of Southern Denmark, 5230 Odense, Denmark; magnim@setur.fo; 6 Centre of Health Sciences, Faculty of Health, University of the Faroe Islands, 100 Tórshavn, Faroe Islands; 7 Department of Computer Science, Faculty of Science and Technology, UiT the Arctic University of Norway, 9037 Tromsø, Norway; ivantm_@hotmail.com

**Keywords:** performance, elite players, match monitoring, team sports, WBGT

## Abstract

Environmental temperature (T_e_) is a main atmospheric parameter that may affect the physical and physiological demands of outdoor sports. Thus, this study aimed to examine the relationship of T_e_ with the physical and physiological demands of beach soccer match-play. Physical and physiological demands were collected from 60 male players during Portuguese elite beach soccer championship matches using a 10 Hz wearable global positioning system, heart rate, and the rate of perceived exertion (RPE). A bilateral counter-movement jump (CMJ) test assessed lower limb power performance before and immediately after the match. Fluid loss was determined by body mass weighing before and after the end of the match. T_e_ and wet-bulb globe temperature (WBGT) parameters were continuously recorded. The matches occurred across T_e_ ranging from ~20.0 °C to 43.0 °C. Physical demands, CMJ height, peak heart rate (HR_peak_), mean heart rate (HR_mean_), and RPE were not correlated with T_e_. However, a significant correlation was found between fluid loss and T_e_ (r [95% CIs] = 0.67 [0.43–0.75]; *p* < 0.001). Beach soccer players maintained physical performance independent of T_e_. The specific characteristics of the sport may have promoted adequate thermoregulatory adaptations, helping maintain the players’ physical performance, particularly in matches played under high T_e_ conditions. Elite beach soccer players maintained their physical performance independently of T_e_ and despite experiencing dehydration (a body mass decrease of more than 2%) when the T_e_ exceeded 35 °C.

## 1. Introduction

The human ability to maintain thermal homeostasis depends on heat exchange with the external environment, which is influenced by atmospheric (temperature, humidity, solar radiation, and wind-speed levels) and behavioral parameters (e.g., metabolism and clothing) [1,2]. The athletic population is not exempt from these influences, particularly in outdoor sports [3], where environmental temperature (T_e_) may affect physical and physiological demands and, consequently, performance and health [3,4,5].

Previous studies have shown that a hot environment (>31.0 °C) is associated with an increased rate of perceived exertion (RPE) [6] and may compromise the ability of athletes to repeat intense exercises [7]. Particularly, at T_e_ > 35.0 °C, aerobic capacity can be negatively affected [4], compromising the effectiveness of specific actions required during competition [8]. For example, soccer players have shown a decreased total distance (TD) covered and high-intensity running (HSR) in matches played at 43.0 °C; however, peak sprinting speed was higher [9]. Moreover, higher maximal power levels at T_e_ > 28.0 °C were observed compared to thermoneutral T_e_ < 21.0 °C [10]. These results suggest a nonlinear relationship between T_e_ and physical performance, whereby warmer T_e_ may be more beneficial for physical performance than thermoneutral T_e_ up to a certain threshold, after which performance may be impaired.

The wet-bulb globe temperature (WBGT) is a thermal stress index that considers T_e_ and other atmospheric parameters such as humidity, solar radiation, and ventilation levels [11]. During a competition, a marked reduction in the running intensity of soccer players was observed under a high environmental stress (WBGT > 28.0 °C) [12]. Playing sports in hot and humid environments is associated with increased body mass loss of > 2% [13], sweat loss, heart rate (HR), core temperature (T_core_), and muscle and skin temperature above values considered normal when exercising in temperate environments [3]. This physiological strain may hamper the thermoregulation process and increase the risk of heat-related illnesses, which can result in withdrawal and/or collapse during or shortly after training and competition [14].

At the 2020 Tokyo Olympic and Paralympic Games (held in 2021), the T_e_ > 30.0 °C and relative humidity ~70% may have resulted in some athletes withdrawing from the competition or needing medical attention [15]. Likewise, T_e_ > 30.0 °C and low humidity have been linked to deleterious effects on the running speed of marathon runners [16] and cardiorespiratory loading of soccer players during submaximal exercise [4]. In the Beach Volleyball World Tour, the risk of heat-related medical time-out increased by 1.8% at T_e_ > 30.1 °C and 2.2% at T_e_ > 32.3 °C [17]. This suggests that the effect of heat on athletes may differ according to T_e_, sport and task specificity [18] beyond the state of hydration, acclimation, and acclimatization [19].

Understanding how athletes respond to different T_e_ is more relevant in outdoor sports, where T_e_ cannot be controlled. In beach soccer, most tournaments are scheduled in the summer, at sea level, and depending on the location, day, and time, matches can occur under different T_e_, typically between 13.0 and 34.0 °C. Moreover, because of the escalating T_e_ in recent years, the risk of playing at high or extreme temperatures (>40.0 °C) is increasing [20]. Additionally, the unstable playing surface, composed of sand, requires significant energy to meet the demands of the intermittent and explosive nature of beach soccer [21,22,23], which may be exacerbated by environmental conditions that increase the risk of thermal stress.

While research about beach soccer players’ physical demands has advanced [21,22,23] physiological responses remain scarce [24,25]. However, these studies indicate that the locomotor and metabolic systems appear to be highly taxed, with mean heart rate (HR_mean_) fluctuating around 74–94% of HR_max_ [21]; however, no impairment in counter-movement jump (CMJ) performance was observed [24]. Nevertheless, these studies did not consider the potential effects of T_e_ and the associated physiological responses, individual characteristics (e.g., anthropometry, fitness level), and sport-specific efforts [26].

Therefore, there is an evidence gap to support coaching and medical staff involved with beach soccer in planning their training based on the information about the influence of temperature on the players’ performance and health. Thus, this investigation aimed to examine the relationship between T_e_ and the physical and physiological demands of beach soccer match-play. It was hypothesized that the players’ physical performance and physiological responses are associated with continuous variations of T_e_, with an increased risk of decline in performance and health T_e_ increases, potentially when exceeding 30.0 °C.

## 2. Materials and Methods

### 2.1. Participants

Sixty male beach soccer players (age: 27.8 ± 6.1 years; height: 180 ± 6 cm; body mass: 73.2 ± 8.8 kg; fat body mass: 8.2 ± 8.8%; lean body mass: 62.6 ± 2.7%; mean ± standard deviation [SD]), from six teams playing at the Portuguese men’s elite championship, were monitored during five official matches. According to standard participant classification previously established criteria [27], the teams were defined as elite (tier 4) because they competed at the senior international level, in the highest national domestic league, and were ranked among the top 100 teams globally [28]. The inclusion criteria encompassed the absence of illness and injuries that inhibited the participants from playing in matches and to active participation in the match for a minimum duration of one minute. All research procedures complied with the Declaration of Helsinki, an updated version of 2013 (Fortaleza), and were approved by the local Ethics Committee (CEFADE.6.2023). The participants read and signed an informed consent form containing a detailed explanation of the risks and potential benefits of the study.

### 2.2. Design

A cross-sectional observational design was adopted [29] to collect environmental conditions, matches, and player data from the official matches. A non-probabilistic convenience sampling method was used based on the availability and willingness of the teams to participate. A post hoc analysis of statistical power was conducted utilizing the G*Power 3.1.9.7 software, employing the bivariate correlation test (normal model). With a sample size of 60 players, an error probability of 0.05 (5%), and an effect size of ρ = 0.25, the statistical power was determined to be 0.61. Four trained evaluators collected data regarding T_e_ and physical and physiological demands from the six teams during four days across five matches played between 23 July and 26 August in the 2023 season. The matches were spaced at intervals ranging from one to three weeks and occurred in the afternoon (between 1 pm and 3 pm), with T_e_ ranging from ~ 20.0 °C to 43.0 °C. Each player engaged in only one match. Players’ height and body mass were collected before the match. The locomotor activity and HR were recorded continuously during the match. The RPE and fluid loss were evaluated after the match. The bilateral countermovement jump (CMJ) was evaluated before and immediately after each match. Match data were examined in relation to T_e_. All the players were previously instructed and familiarized with using the instruments during one training session conducted in the week of each examined match.

### 2.3. Data Collection

#### 2.3.1. Environmental Conditions

T_e_ and WBGT were measured at two-minute intervals [30] during each match using a portable climate monitoring device (Kestrel 5400 Heat Stress Tracker, Nielsen-Kellerman, Boothwyn, PA, USA) with manufacturer-reported accuracy of ±0.7 °C. The device was powered 15 min before each match to allow the device to equilibrate to atmospheric conditions and positioned over a tripod, at a 1.2 m height, adjacent to the match to capture environmental conditions [31].

#### 2.3.2. Physical Demands

Match physical demands were recorded using a 10 Hz wearable technology (WIMU PRO^TM^; RealTrack Systems, Almeria, Spain). These devices incorporate different tracking technologies, including the 10 Hz global positioning system (GPS) with validity (bias in mean velocity 1.2–1.3 km/h; bias in distance 2.3–4.3 m) and reliability (ICC > 0.93) previously reported in team sport athletes [32], and 100 Hz accelerometer which is reliable and widely used in sports science research [33]. The devices were turned on approximately 30 min before the match according to the manufacturer’s recommendations and placed in a specific custom vest between the scapulae of each player. At the end of the matches, the inertial devices were removed from the players and the data were downloaded and analyzed through specific software S-PRO^TM^ version 1.0.0 (RealTrack Systems, Almería, Spain).

Physical data were coded into the following categories and speed thresholds, which were also used in previous beach soccer studies: HSR (13.0–17.9 km·h^−1^), number of sprints (>18 km·h^−1^), TD and peak speed (*V*_peak_) [25]. The distance covered by acceleration > 3.0 m·s^−2^ and deceleration < −3.0 m·s^−2^ were also quantified [34].

Before and after lower limb power performance was assessed to examine match-induced fatigue through the CMJ test on a stable surface. CMJ height was estimated using an optical analysis and measurement system (Optojump, Microgate, Bolzano, Italy). The absolute difference between the measurements made after and before the matches was calculated to quantify the neuromuscular fatigue. This device is strongly associated with gold-standard measurements (r = 0.98) and good test–retest reliability (ICC = 0.97) [35]. The players were instructed to (i) descend from a standing position, flex their knees at a freely chosen angle, and perform a vertical jump as high as possible, (ii) keep their hands on the waist throughout the movement, (iii) repeat these movements two times with 30 s of rest between attempts. The highest trial was retained for the analyses [7,24].

#### 2.3.3. Physiological Outcomes

##### Heart Rate

Physiological demands were recorded using a short-range telemetry system with a 5 s interval through GARMIN™ bands (Garmin Ltd., Olathe, KS, USA) synchronized with the GPS software WIMU PRO™ version 1.0.0 (RealTrack Systems SL, Almeria, Spain). This system has shown reasonable validity in measuring HR during exercise, based on a robust correlation in relation to a previously validated measurement device (*r*^2^ = 0.96) [36]. Measurements were taken externally through an electrode belt strapped around the chest at the lower sternum of each player before the start of each match. The procedures followed the manufacturer’s recommendations, and the data were downloaded after each match using the software S-PRO^TM^ version 1.0.0 (RealTrack Systems, Almería, Spain).

##### Rate of Perceived Exertion

The RPE was collected using the Borg category ratio scale (CR-10) [37]. The CR-10 is a category-ratio scale of 0 to 10 (with 0 being rest and 10 being maximal effort) that asks users to rate their perception of physical effort in relation to a task [38]. Thus, the players answered the question: “How would you rate the intensity of the match?” approximately 30 min after each match to ensure that the perceived effort was referred to the whole match rather than the last action’s intensity performed in the match [39]. The players reported their RPE individually to avoid being influenced by other teammates’ responses.

##### Fluid Loss

The amount of fluid loss was determined after voiding by body mass weighing before and after the match. The fluid intake during the match was controlled by weighing individually labeled bottles. The players were instructed (i) to drink only from their own numbered bottles and not spit, (ii) not to discard any drinks, and (iii) to dry themselves before weighing. As body composition technology cannot be used during sweating (due to temporary heat-induced changes in conductivity within the body), we tacked a conservative approach based on a digital scale (Seca 813, Hamburg, Germany) [40].

### 2.4. Statistical Analyses

The Kolmogorov–Smirnov test revealed that match data were normally distributed, except for the number of sprints. The associations between T_e_ and physical demands data and physiological response were quantified using Pearson’s correlation (r), while Spearman’s correlation coefficient was employed for the number of sprints, and respective confidence intervals (95% CIs) as follows: ≤0.1, trivial; 0.1–0.3, small; >0.3–0.5, moderate; >0.5–0.7, large; >0.7–0.9, very large; and >0.9–1.0, almost perfect [41]. Bonferroni correction was performed to minimize the risk of type I error: the usual 0.05 *p*-value for statistical significance was divided by the number of statistical tests and set at 0.004 [42]. Data are expressed as mean and SD. All analyses were performed in IBM SPSS software version 27.0 (Chicago, IL, USA).

## 3. Results

### 3.1. Environmental Conditions

During the matches, T_e_ and WBGT varied from 24.4 °C to 43.1 °C (30.0 ± 4.9 °C) and 22.0 °C to 33.0 °C (26.0 ± 2.7), respectively (Table 1). An almost perfect correlation (r [95% CIs] = 0.96 [0.93–0.98]; *p* < 0.001) was observed between T_e_ and WBGT; therefore, we decided to interpret the results based on T_e_.

### 3.2. Physical Demands

Beach soccer players covered (overall mean ± standard deviation) a TD of 1936 ± 606 m, of which 182 ± 95 m were performed at high intensity. The V_peak_ attained was 20.0 ± 1.9 km/h, with a number of sprints of ~4.0 ± 3.5. The mean distances covered during accelerations > 3.0 m·s^−2^ and decelerations < −3.0 m·s^−2^ were 88 ± 50 m and 99 ± 52 m, respectively (Table 2).

**Figure 1:**
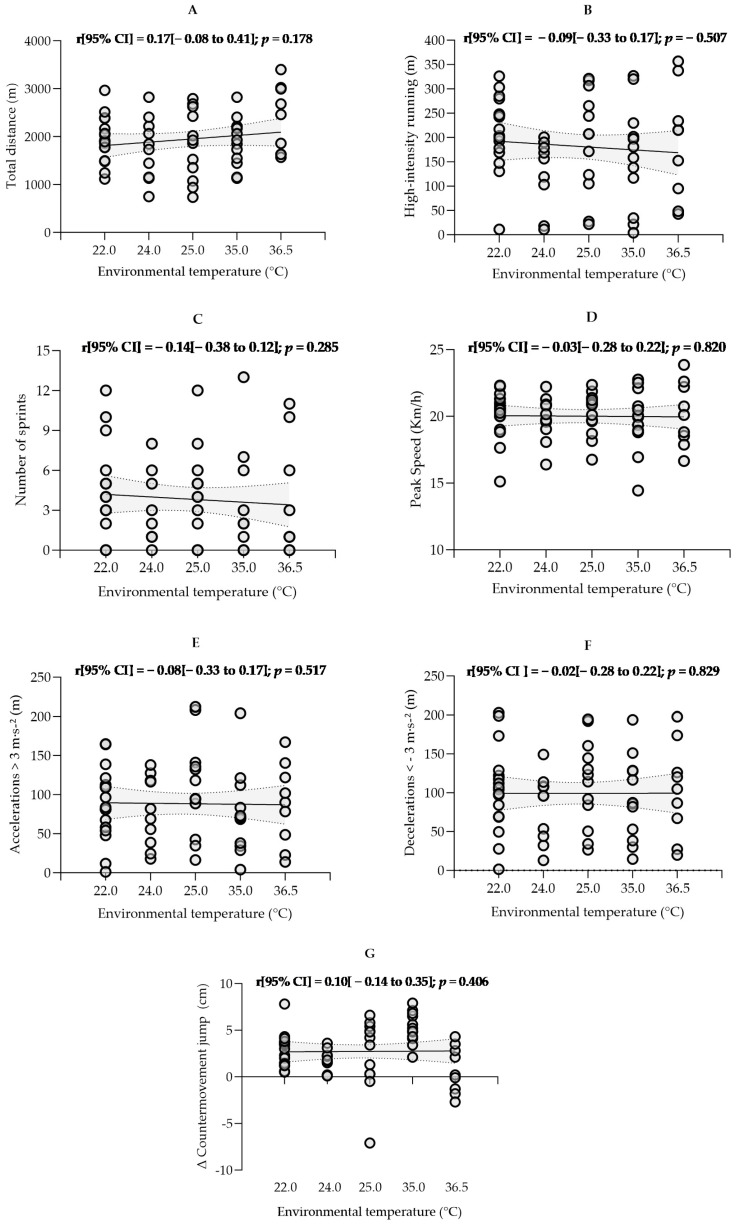
Spearman’s correlation for the (**C**) number of sprints performed and Pearson’s correlation for the remaining variables between the environmental temperature and physical demands: (**A**) Total distance covered, (**B**) Distance in high-intensity running, (**D**) Peak speed attained, (**E**) Distances covered during accelerations, (**F**) Distances covered during deceleration, and (**G**) Absolute difference between bilateral counter-movement jump after and before the matches. 95%CI, 95% confidence interval.

No significant correlations were observed between different T_e_ conditions and the players’ locomotor activity variables (*p* > 0.004; Figure 1). CMJ performance increased after the match under different T_e_ but decreased when exceeded 36.0 °C (Table 2). However, the Pearson correlation did not indicate an association between T_e_ and CMJ (r = 0.10; Figure 1G). This suggests that the relationship may not be linear or that T_e_ does not negatively affect the CMJ within a certain range.

**Figure 2:**
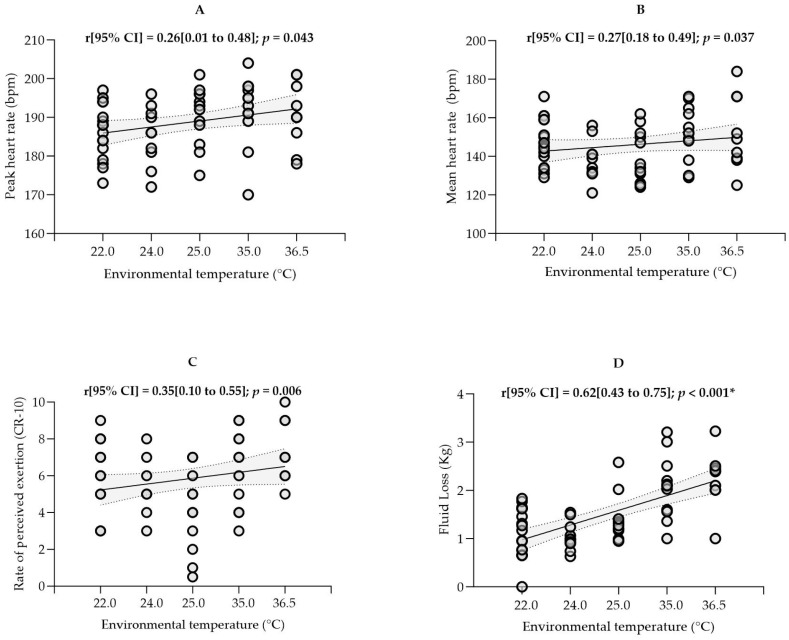
Pearson’s correlation between environmental temperature and physiological outcomes: (**A**) Maximum heart rate attained, (**B**) Mean heart rate attained, (**C**) Rate of perceived exertion, and (**D**) Fluid loss during the match. 95%CI, 95% confidence interval; CR-10, Borg category ratio scale of 0 to 10. * *p* ≤ 0.004.

Nevertheless, our results show considerable inter-individual variability in physical and physiological demands, especially in the number of sprints (Figure 1C) and RPE (Figure 2C).

### 3.3. Physiological Outcomes

The players’ HR_peak_ and HR_mean_ were 188.7 ± 8.0 bpm and 145.8 ± 14.4 bpm, respectively, along with a RPE of 6.0 ± 2.0. No significant correlations were observed between the different T_e_ conditions and these variables (Figure 2), although the players reported greater effort in matches where T_e_ exceeded 35 °C (7.0 ± 1.5).

Fluid intake was 1.2 ± 0.3 L and 1.3 ± 0.5 L when the matches were played at higher T_e_ (35.0 °C and 36.5 °C) and 0.8 ± 0.3 L, 0.8 ± 0.2 L, and 1.0 ± 0.4 L at milder T_e_ (22.0, 24.0, and 25.0 °C, respectively). Regarding fluid loss, at higher T_e_ (35.0 and 36.5 °C), the players’ body mass decreased by ~2.1 kg after the match, corresponding to a net loss of ~2.0 L (>2.7% body mass). While at T_e_ of 22.0, 24.0, and 25.0 °C, the body mass loss was ~1.0 ± 0.6 kg, 1.0 ± 0,3 kg, and 1.3 ± 0.4 kg, respectively. A large correlation was observed between T_e_ and fluid loss (r [95%CI] = 0.62 [0.43–0.75]; *p* < 0.001; Figure 2D).

## 4. Discussion

To our knowledge, this is the first study to examine the relationship between T_e_ and the physical and physiological demands of elite beach soccer players in a real-world competitive scenario. This is even more relevant in light of climate change and the fact that beach sports are often practiced during the summer months, exposing athletes to the risk of competing in T_e_ > 40.0 °C, which is expected to not only impair performance but also contribute to the development of heat-related illnesses [14,20]. The matches observed were played during the middle of the season (July to August) of the Portuguese men’s elite championship, with temperatures ranging from 20.4 °C to 43.1 °C. Although we hypothesized an increased risk of decline in performance and health with T_e_ increases, our results allude that beach soccer players’ physical and physiological demands are unrelated to T_e_. However, players lost a greater amount of fluid when playing at higher T_e_ (r = 0.60).

Importantly, the considerable inter-individual variability observed in physical demands and RPE may have influenced the relationship analyzed. Individual factors, such as fitness level, acclimatization to heat, and positional demands within the team, may play a crucial role in the players’ responses to the T_e_ conditions. A recent study found that wingers experience greater physical demands and engage in more high-intensity actions compared to players in other positions [22]. Consequently, it is possible that these players are more affected by high T_e_ [7] compared to goalkeepers, whose physical demands during the match are significantly lower [22].

### 4.1. Physical Demands

Beach soccer players managed to maintain a consistent HSR and *V*_peak_ across a range of temperatures differing by ~15.0 °C. In matches under high T_e_, players may conceivably manage their physical efforts to preserve their ability to perform high-intensity activities at critical moments, as previously observed in other sports [6,12,17,43]. In our study, the number of sprints was greater at 22.0 °C, while the highest values of TD were observed at 36.5 °C, but with no relationship to T_e_. Also, as previously shown in soccer, a high muscle temperature in the heat may favor high muscle contraction velocities that may partly counteract the adverse effects of hyperthermia and dehydration [9].

Our findings differed from those observed in other team sports in a competitive context. For example, in field hockey, HSR was compromised when the temperature exceeded 25.0 °C [44]. In soccer, the TD covered by players during matches in the top German soccer leagues decreased as T_e_ increased, from 14.0 °C onwards, from −10.0 °C to 28.0 °C [43]. Conversely, studies using categorical WBGT values revealed no effect on the aerobic capacity of soccer players in hot climates (WBGT 24.0 °C to 33.0 °C), compared to levels < 24.0 °C, whereas anaerobic capacity was negatively affected [12,45]. However, in these studies, environmental conditions were measured only once, one hour before the start of each match, following the FIFA standards. Thus, meaningful information about the variation in environmental conditions throughout the match may have been lost.

The difference in CMJ performance before and after matches was unrelated to T_e_. The CMJ height increased after the matches played at thermoneutral T_e_ and 35.0 °C. Previously, a 7% increase in jump height was reported in beach soccer players, even without player rotation [24]. However, no information was provided regarding the environmental conditions under which the match occurred, making it difficult to compare with our data. In the match played under 36.5 °C, we observed a slight reduction in the height of the CMJ after the match, which may indicate a threshold at which the lower limb power begins to be impaired. In soccer players, the mean height-repeated CMJ declines by about 9% after the match was played already at T_e_ ~ 31.0 °C [7]. Therefore, it is advisable to examine further matches played under different T_e_ > 30.0 °C to verify if the continuous increase in T_e_ harms the neuromuscular status of beach soccer players so that more accurate conclusions can be drawn.

Regardless, the heterogeneity between the studies suggests that physical responses to varying T_e_ may not follow a universal pattern, as individual and specific characteristics of each sport, and their nuances [23], may influence how the players respond to different T_e_. In beach soccer, the shorter playing time, unlimited substitution regulations, and the rest between periods [22] may contribute to maintaining performance, even in adverse environmental conditions.

### 4.2. Physiological Outcomes

Our results indicate that HR and RPE were unrelated to T_e_, although we observed a tendency for the RPE to increase from 35.0 °C onwards. Regardless of T_e_, the players maintained the intensity in performing the required tasks during the matches, as evidenced by the HSR. It is possible that the hotter T_e_ did not increase the players’ HR due to unlimited rotation throughout the match, which is a feature of beach soccer that allows for frequent rest periods and hydration opportunities. These findings differ from those reported in other sports, where an increase in HR [8] and RPE [6] was observed with subsequent impairment in athletes’ performance during matches played in T_e_ > 31.0 °C.

In our study, players consumed a mean of ~1 L of fluid, with a maximum of 1.5 L during warmer T_e_ matches. This hydration level may have contributed to increased HR and RPE, partially counterbalancing the increase in body mass fluid loss as T_e_ increased (deficit of ~3% of initial body mass after matches played at T_e_ > 35.0 °C). During exercise in the heat, a body mass loss of >2% can harm health and athletic performance [13], as evidenced by studies with soccer players in simulated, training, and competitive contexts [7,9,46]. However, the beach soccer players in this study did not experience any impairment in physical and physiological demands, which may also be attributed to their consistent and progressively increasing fluid intake that matched the increase in T_e_. However, repeated fluid loss greater than 2% of body mass can result in adverse cumulative effects, including an increased risk of developing heat-related illnesses [14], impaired cognition and physical performance [47], especially in summer sports commonly practiced in high T_e_, such as beach soccer, which demands frequent high-intensity efforts [21,22]. These effects may be particularly concerning in pre-season tournaments with multiple matches per day, as players are unlikely to have acclimated, fitness is still developing, and recovery between the matches is brief, thereby increasing the players’ vulnerability to thermal stress and dehydration.

The matches occurred in the middle of the season, so it is conceivable that the players had undergone a process of seasonal acclimatization. That is, exposure to high T_e_ during training and the first months of competition may have contributed to their heat tolerance [14]. Evidence suggests that even one week of acclimatization can already lead to improvements in players’ cardiovascular capacity and neuromuscular function [48]. Improvements to thermoregulatory mechanisms were observed in soccer players [49], such as increased sweat production, improved blood flow to the skin, and expansion of plasma volume, which may have contributed to the dissipation of metabolic heat and consequent maintenance of HR at ideal levels and the health of players during matches that were played in hot T_e_ [19]. Furthermore, in matches where T_e_ was > 30.0 °C, relative humidity remained close to 30% (Table 1); this dry-heat condition may have facilitated more effective body evaporative cooling, potentially reducing cardiovascular strain [3]. However, future research that directly compares the differences observed at the beginning, middle, and end of the season is required to better understand these adaptations, particularly in the context of beach invasion sports [23].

### 4.3. The Potential Influence of Statistical Analyses

As suggested by the specialized literature [42,50], we applied a Bonferroni correction to the *p*-value to account for multiple statistical tests. Although this statistical procedure is necessary to minimize the probability of type I error, its application in sports science is still limited [51]. Without such statistical correction, our study would have presented additional statistically significant correlations (Figure 1 and Figure 2), as the *p*-value would have remained 0.05 instead of 0.004. To our knowledge, comparable studies [12,44] did not perform such family-wise error rate corrections. Thus, potential significant effects observed in previous studies may represent a statistical artifact.

### 4.4. Limitations

Despite the importance of our findings, the number of observed matches (five) was low, restricting the opportunity to comprehensively analyze physical and physiological demands across a wider range of different T_e,_ to enhance the scope of the preliminary conclusions of this study. Understanding how an increase or decrease of 1.0 °C could influence the players’ performance would be enlightening. T_core_ monitoring was intended to be conducted through an ingestible pill thermometer. Given the high cost of the pills and the players’ reluctance to ingest them, this proved unfeasible. Thus, less invasive and affordable equipment is required to monitor T_core_ so that, in conjunction with thermal sensation and comfort measurements, we can better understand the relation of T_e_ on thermoregulatory and perceptual responses. Furthermore, CMJ height may mask acute neuromuscular fatigue due to individual variations and neuromuscular adaptations of players during the match [52].

The statistical analyses conducted allowed the correlation coefficients to assess the strength and direction of associations, but they did not imply causality. Additionally, the Bonferroni correction was applied to minimize the risk of Type I errors (false positives); however, this approach may have increased the likelihood of Type II errors (false negatives), thereby reducing sensitivity in detecting potential associations [42].

Also, we acknowledge that team sports are inherently random in nature, and factors such as match-to-match variability, match importance, opposition, coaching strategies, tactical approaches, individual fitness levels, effective playing time, intensity of physical effort associated with position of the players, sand variability, and other external variables cannot be disregarded. Furthermore, we did not control the hydration status of players before matches, which may have influenced both the need for fluid intake and the magnitude of fluid loss during the matches. Although a correlation between fluid loss and T_e_ was observed, the combined relationship between fluid loss, fluid intake, and physical demands was not examined, which limits the understanding of their potential effects on players’ performance.

Therefore, future investigations should consider these factors in studies on larger sample sizes (encompassing multiple teams, players, and matches) conducted across a wide range of T_e_ conditions, and with monitoring players throughout the season. These studies may provide more specific insights and contribute to a deeper understanding of the effects of T_e_ on players’ performance.

## 5. Conclusions

Elite beach soccer players appear to maintain their physical performance regardless of T_e_, despite experiencing dehydration when the T_e_ exceeded 35 °C. Possibly, acclimatization, dry-heat conditions, ample opportunities for rest (due to unlimited substitutions), and continuous fluid intake may have facilitated adequate body temperature regulation, particularly in matches under high T_e_ conditions.

## 6. Practical Applications

The regulations and characteristics of beach soccer offer frequent opportunities for hydration; therefore, coaching and medical staff should raise awareness, educate, and encourage players to appropriately hydrate throughout the match, mainly in hot conditions. Additionally, coaches can devise and optimize strategic player rotations for use in matches under high T_e_ to mitigate heat-related illnesses and maintain the team’s high overall physical performance throughout the match.

## Figures and Tables

**Table 1:** Mean ± standard deviation (SD), minimum (Min), and maximum (Max) values of environmental conditions by match.

		T_e_ (°C)			WBGT (°C)			RH (%)		
Match	Players (*n*)	Min	Max	Mean ± SD	Min	Max	Mean ± SD	Min	Max	Mean ± SD
1	17	20.4	24.5	22.0 ± 1.1	22.1	24.6	23.7 ± 0.6	66.7	80.5	74.9 ± 3.7
2	12	23.0	29.9	25.0 ± 1.5	24.0	25.5	24.6 ± 0.4	61.6	74.7	70.1 ± 2.1
3	9	32.6	43.1	36.5 ± 2.8	29.7	32.9	31.0 ± 0.6	25.6	39.0	33.3 ± 3.1
4	12	31.5	38.3	35.0 ± 1.9	28.5	31.6	29.0 ± 0.7	28.0	35.0	31.2 ± 1.9
5	10	23.8	25.8	24.0 ± 0.5	25.7	27.7	26.4 ± 0.4	57.3	67.0	61.9 ± 3.0
Overall	60	20.4	43.1	28.5 ± 6.7	22.1	32.9	26.9 ± 2.7	25.6	80.5	54.3 ± 18.4

Abbreviations: T_e_, Environmental temperature; WBGT, wet-bulb globe temperature; RH, relative humidity.

**Table 2:** Mean ± standard deviation (SD) players’ physical demands and physiological responses during matches with different environmental temperatures (T_e_).

T_e_		22.0 °C	24.0 °C	25.0 °C	35.0 °C	36.5 °C
Players (*n*)		17	12	10	12	09
**Physical demands**						
HSR (m)		213 ± 73	134 ± 66	193 ± 101	161 ± 101	189 ± 108
Number of sprints		4.5 ± 3.2	2.8 ± 2.5	4.5 ± 3.1	3.3 ± 3.5	3.5 ± 4.1
Peak speed (Km/h)		20.1 ± 1.7	19.8 ± 1.6	20.1 ± 1.6	19.7 ± 2.2	20.1 ± 2.2
Total distance (m)		1940 ± 453	1756 ± 611	1830 ± 681	1871 ± 483	2361 ± 665
Accelerations (m·s^−2^)		89 ± 45	79± 42	110 ± 60	76 ± 50	87 ± 49
Decelerations (m·s^−2^)		104 ± 52	81 ± 41	112 ± 54	92 ± 51	102 ± 57
CMJ (cm)	Before	35.7 ± 5.2	38.9 ± 6.1	37.6 ± 4.5	35.1 ± 5.0	38.0 ± 4.3
	After	38.2 ± 5.8	40.6 ± 6.1	40.0 ± 6.9	40.3 ± 5.9	38.2 ± 4.7
**Physiological responses**						
HR_peak_ (bpm)		186.2 ± 6.6	185.3 ± 7.2	190.4 ± 7.2	191.8 ± 8.5	190.7 ± 8.1
HR_mean_ (bpm)		147.2 ± 11.6	137.9 ± 10.0	139.7 ± 12.8	151.7 ± 13.5	152.3 ± 18.0
RPE (CR-10)		5.9 ± 1.5	5.3 ± 1.3	4.1 ± 2.0	6.6 ± 2.0	7.3 ± 1.5
Fluid Loss (Kg)		1.0 ± 0.5	1.0 ± 0.3	1.3 ± 0.4	2.0 ± 0.6	2.2 ± 0.8

Abbreviations: T_e_, environmental temperatures; HSR, high-intensity running; CMJ, countermovement jump height; HR_peak_, peak heart rate and HR_mean,_ mean heart rate; RPE, rate of perceived exertion; CR-10, Borg category ratio scale of 0 to 10.

## Data Availability

The data supporting the conclusion of this study are available openly available in the Open Science Framework (OSF) at https://osf.io/zmnw9/?view_only=cf4655692631432b8291cf6fa9813a0e (accessed on 6 December 2024).

## Abbreviations

The following abbreviations are used in this manuscript:

T_e_	Environmental temperature
RPE	Rate of perceived exertion
HSR	High-intensity running
TD	Total distance
*V*_peak_	Peak speed
WBGT	Wet-bulb globe temperature
HR	Heart rate
HR_peak_	Peak heart rate
HR_mean_	Mean heart rate
CMJ	Counter-movement jump
GPS	Global positioning system
